# Special Issue “Oral Cancer and Disease in Humans and Animals”

**DOI:** 10.3390/ijms27020810

**Published:** 2026-01-14

**Authors:** Leonor Delgado, Luís Monteiro

**Affiliations:** 1 INNO Veterinary Laboratories, R. Cândido de Sousa 15, 4710-300 Braga, Portugal; 2 Department of Veterinary and Animal Sciences, University Institute of Health Sciences (IUCS), CESPU, 4585-116 Gandra, Portugal; 3 UNIPRO-Oral Pathology and Rehabilitation Research Unit, University Institute of Health Sciences-CESPU (IUCS-CESPU), 4585-116 Gandra, Portugal; luis.monteiro@iucs.cespu.pt; 4 GIPOC-Comparative Oral Pathology Research Group, University Institute of Health Sciences-Advanced Polytechnic and University Cooperative (IUCS-CESPU), 4585-116 Gandra, Portugal

Oral cancer represents a major cause of death mainly due to the existence of important gaps in the knowledge of and subsequent interventions in these neoplasms, including many factors, such as the late diagnosis of neoplastic lesions and a lack of treatment options, that contribute to the poor survival of these patients [1,2,3].

Its development is strongly associated with several well-established risk factors. Tobacco use and excessive alcohol consumption are the most significant contributors, not forgetting the role of inhalation of secondhand smoke [4,5]. The study of premalignant lesions of the oral cavity, such as leukoplakia and erythroplakia, is of great importance, as these conditions may represent early stages in the progression to oral cancer [6,7].

From this point of view, the molecular study of these lesions and the search for molecules with prognostic value (cancer biomarkers, molecular diagnostics, immunotherapy, etc.) can open doors for a specialized characterization of each human or companion animal patients and better prognoses [8,9,10,11].

This edition highlights innovative studies, critical reviews and a case report that encompass advances in molecular characterization of oral cancer, mainly oral squamous cell carcinoma and potentially malignant oral disorders, with strong implications for clinical decision making.

We sincerely thank the authors, reviewers, and editorial team, whose contributions made this Special Issue a reality. Their work demonstrates both the depth of scientific research and the teamwork required to address a major challenge in oncology field.

One review in this issue by Prime et al. investigates how failures in repairing DNA double-strand breaks contribute to the emergence of oral squamous cell carcinoma [4]. The authors highlight that defects within the homologous recombination and Fanconi anaemia pathways are especially critical in transforming potentially malignant oral disorders into invasive cancer. By comparing mortal and immortal keratinocyte cell lines, the study demonstrates that the overexpression of specific repair genes correlates with genomic instability and the bypass of cellular senescence. Evidence from familial conditions and cases involving younger patients suggests that these genetic abnormalities are pivotal, even in the absence of traditional risk factors like tobacco. Ultimately, maintaining telomere homeostasis and effective DNA repair is fundamental to preventing oral malignancy.

Murthy et al. performed a second review that systematically examines the role of keratins as potential biomarkers in the diagnosis and management of oral leukoplakia, a condition often preceding cancer. By analysing over forty original studies, the authors identify critical patterns such as the loss of primary keratins (like K13 and K10) and the atypical gain of expression in others (like K17 and K19). A significant shift from normal cell structure to dysplastic disruption suggests these protein changes could eventually help clinicians distinguish between low-risk and high-risk lesions. Ultimately, the research aims to bridge the gap between complex molecular pathology and practical clinical tools.

New research is presented in this Special Issue, such as a study by de Vicente et al. investigating the influence of the NOTCH1 signaling pathway on the progression and clinical outcomes of oral squamous cell carcinoma (OSCC). By studying 165 patient samples, the authors discovered that NOTCH1 protein expression acts as a significant independent predictor of poor survival, correlating with advanced cancer stages and metastasis. In contrast, downstream markers like HES1 and p21 were associated with earlier stages of the disease and better patient prospects. The study highlights a vital link between p21 and mTORC1 activation, suggesting this combination could serve as a reliable tool for identifying low-risk cases. Ultimately, the findings suggest that while NOTCH1 may drive aggressive tumor behavior, its regulatory role is complex and often influenced by other independent molecular pathways.

Also a research article by Ivkovic et al. investigates how specific immune-related genes and their genetic variants influence the progression of HPV-negative oral squamous cell carcinoma. By comparing a primary patient cohort with larger validation datasets, the authors identify FOXP3 and miR-155 as significant prognostic biomarkers for patient outcomes. Specifically, low FOXP3 expression is strongly linked to shorter survival and more advanced disease, while high miR-155 levels correlate with increased cancer recurrence. The study further highlights a non-linear relationship between gene activity and tumor stage, suggesting that immune responses fluctuate as the disease worsens. Ultimately, these findings suggest that integrated immune profiling could help clinicians better predict survival and tailor immunotherapy treatments for oral cancer patients.

Sanjuan-Sanjuan et al. investigated how the splicing machinery, which regulates protein diversity in cells, is significantly dysregulated in oral squamous cell carcinoma. By comparing patient tumours with healthy tissue, the authors identified twelve splicing factors that are altered, many of which correlate with clinical outcomes such as survival rates and tumour aggressiveness. These findings suggest that specific components of the spliceosome could serve as vital diagnostic and prognostic biomarkers for oral cancer patients. Additionally, the study demonstrates that using a pharmacological inhibitor to target this machinery effectively reduces the proliferation of cancer cells in laboratory cultures. Ultimately, the work highlights the therapeutic potential of targeting splicing processes as a novel strategy for treating this malignancy.

And finally, a research article by Palmieiri et al. explores the biological mechanisms behind drug-induced gingival overgrowth (DIGO), a condition where certain medications cause excessive gum tissue expansion. The study specifically examines how anticonvulsants, immunosuppressants, and calcium channel blockers affect the behaviour of macrophages, which are essential immune cells. By exposing human cells to these drugs in a laboratory setting, researchers discovered that the medications trigger a shift towards a pro-inflammatory M1 phenotype. This cellular transformation involves the upregulation of specific genes such as IDO1, CXCL10, and CCL5. The findings suggest that this drug-induced inflammation may be a primary driver of the fibrosis and tissue swelling seen in patients. Consequently, targeting this macrophage polarization could offer a new therapeutic pathway for managing the side effects of these chronic medical treatments.

Equally fascinating is the case report by Alonso-Juarranz and his colleagues of a rare malignant transformation of a squamous odontogenic tumor (SOT). While SOTs are typically classified as benign epithelial lesions of the jaw, this report documents an exceptional instance where the tumor evolved into invasive squamous cell carcinoma in an elderly patient. The researchers utilised massive DNA sequencing to identify specific pathogenic mutations in genes such as RET, POLE, and BRCA2, which likely influenced the tumor’s aggressive clinical progression. By combining histopathological analysis with molecular profiling, the authors aim to improve the diagnostic accuracy and personalised treatment strategies for this infrequent disease.

We hope that the findings and insights presented in this first Special Issue will inspire further and deeper research, leading to a better understanding of the biology and oncogenesis of oral cancer, thereby promoting innovation and collaboration between disciplines, particularly human and veterinary medicine, and contributing to the development of treatments and a better prognosis.

## References

[1] Warnakulasuriya S., Kujan O., Aguirre-Urizar J.M., Bagan J.V., González-Moles M.Á., Kerr A.R., Lodi G., Mello F.W., Monteiro L., Ogden G.R. (2021). Oral potentially malignant disorders: A consensus report from an international seminar on nomenclature and classification, convened by the WHO Collaborating Centre for Oral Cancer. Oral Dis..

[2] Monteiro L.S., Antunes L., Santos L.L., Bento M.J., Warnakulasuriya S. (2018). Survival probabilities and trends for lip, oral cavity and oropharynx cancers in Northern Portugal in the period 2000–2009. Ecancermedicalscience.

[3] Noguti J., De Moura C.F., De Jesus G.P., Da Silva V.H., Hossaka T.A., Oshima C.T., Ribeiro D.A. (2012). Metastasis from oral cancer: An overview. Cancer Genom. Proteom..

[4] Chamoli A., Gosavi A.S., Shirwadkar U.P., Wangdale K.V., Behera S.K., Kurrey N.K., Kalia K., Mandoli A. (2021). Overview of oral cavity squamous cell carcinoma: Risk factors, mechanisms, and diagnostics. Oral Oncol..

[5] Mariano L.C., Warnakulasuriya S., Straif K., Monteiro L. (2022). Secondhand smoke exposure and oral cancer risk: A systematic review and meta-analysis. Tob Control..

[6] Prostakishina E.A., Sidenko E.A., Kolegova E.S., Patysheva M.R., Kononova G.A., Choinzonov E.L. (2025). Premalignant lesions of the oral cavity: A narrative review of factors and mechanisms of transformation into cancer. Int. J. Oral Maxillofac. Surg..

[7] Warnakulasuriya S. (2018). Clinical features and presentation of oral potentially malignant disorders. Oral Surg. Oral Med. Oral Pathol. Oral Radiol..

[8] Monteiro L., Mariano L.C., Warnakulasuriya S. (2024). Podoplanin could be a predictive biomarker of the risk of patients with oral leukoplakia to develop oral cancer: A systematic review and meta-analysis. Oral Dis..

[9] Lenouvel D., González-Moles M.Á., Ruiz-Ávila I., Chamorro-Santos C., González-Ruiz L., González-Ruiz I., Ramos-García P. (2021). Clinicopathological and prognostic significance of PD-L1 in oral cancer: A preliminary retrospective immunohistochemistry study. Oral Dis..

[10] Silva P.M.A., Delgado M.L., Ribeiro N., Florindo C., Tavares Á.A., Ribeiro D., Lopes C., do Amaral B., Bousbaa H., Monteiro L.S. (2019). Spindly and Bub3 expression in oral cancer: Prognostic and therapeutic implications. Oral Dis..

[11] Delgado L., Brilhante-Simões P., Garcez F., Monteiro L., Pires I., Prada J. (2022). p-S6 as a Prognostic Biomarker in Canine Oral Squamous Cell Carcinoma. Biomolecules.

